# Differential diagnosis of parkinsonism: a head-to-head comparison of FDG PET and MIBG scintigraphy

**DOI:** 10.1038/s41531-020-00141-y

**Published:** 2020-12-11

**Authors:** Joachim Brumberg, Nils Schröter, Ganna Blazhenets, Lars Frings, Jens Volkmann, Constantin Lapa, Wolfgang H. Jost, Ioannis U. Isaias, Philipp T. Meyer

**Affiliations:** 1 Department of Nuclear Medicine, University Hospital and Julius-Maximilian-University Würzburg, Würzburg, Germany; 2 Faculty of Medicine, Department of Neurology, Medical Center–University of Freiburg, University of Freiburg, Freiburg im Breisgau, Germany; 3 Berta-Ottenstein-Programme for Clinician Scientists, Faculty of Medicine, University of Freiburg, Freiburg im Breisgau, Germany; 4 Faculty of Medicine, Department of Nuclear Medicine, Medical Center–University of Freiburg, University of Freiburg, Freiburg im Breisgau, Germany; 5 Department of Neurology, University Hospital and Julius-Maximilian-University Würzburg, Würzburg, Germany; 6 Department of Nuclear Medicine, University Hospital Augsburg, Augsburg, Germany; 7 Parkinson-Klinik Ortenau, Wolfach, Germany

**Keywords:** Neurodegenerative diseases, Movement disorders, Parkinson's disease

## Abstract

[^18^F]fluorodeoxyglucose (FDG) PET and [^123^I]metaiodobenzylguanidine (MIBG) scintigraphy may contribute to the differential diagnosis of neurodegenerative parkinsonism. To identify the superior method, we retrospectively evaluated 54 patients with suspected neurodegenerative parkinsonism, who were referred for FDG PET and MIBG scintigraphy. Two investigators visually assessed FDG PET scans using an ordinal 6-step score for disease-specific patterns of Lewy body diseases (LBD) or atypical parkinsonism (APS) and assigned the latter to the subgroups multiple system atrophy (MSA), progressive supranuclear palsy (PSP), or corticobasal syndrome. Regions-of-interest analysis on anterior planar MIBG images served to calculate the heart-to-mediastinum ratio. Movement disorder specialists blinded to imaging results established clinical follow-up diagnosis by means of guideline-derived case vignettes. Clinical follow-up (1.7 ± 2.3 years) revealed the following diagnoses: *n* = 19 LBD (*n* = 17 Parkinson’s disease [PD], *n* = 1 PD dementia, and *n* = 1 dementia with Lewy bodies), *n* = 31 APS (*n* = 28 MSA, *n* = 3 PSP), *n* = 3 non-neurodegenerative parkinsonism; *n* = 1 patient could not be diagnosed and was excluded. Receiver operating characteristic analyses for discriminating LBD vs. non-LBD revealed a larger area under the curve for FDG PET than for MIBG scintigraphy at statistical trend level for consensus rating (0.82 vs. 0.69, *p* = 0.06; significant for investigator #1: 0.83 vs. 0.69, *p* = 0.04). The analysis of PD vs. MSA showed a similar difference (0.82 vs. 0.69, *p* = 0.11; rater #1: 0.83 vs. 0.69, *p* = 0.07). Albeit the notable differences in diagnostic performance did not attain statistical significance, the authors consider this finding clinically relevant and suggest that FDG PET, which also allows for subgrouping of APS, should be preferred.

## Introduction

Radionuclide or molecular imaging techniques support the differential diagnosis of parkinsonism^1,2^ and have been incorporated into current international diagnostic criteria^3–6^. The recommendations refer to both [^18^F]fluorodeoxyglucose (FDG) PET as well as [^123^I]metaiodobenzylguanidine (MIBG) scintigraphy for the differential diagnosis in uncertain clinical cases of suspected neurodegenerative parkinsonism. The selection of a specific technique relies on the particular clinical presentation and question (see guidelines, e.g. pathological MIBG scintigraphy as supportive criterion for Parkinson’s disease [PD] or degeneration of putamen on FDG PET as a feature of possible multiple system atrophy [MSA]).

FDG PET is used to image regional cerebral glucose metabolism as a marker of neuronal activity that may be altered through neurodegeneration and disease-specific network changes. The identification of cerebral metabolic patterns related to specific neurodegenerative diseases^7^ allows to separate Lewy body diseases (LBD; i.e. PD, PD dementia [PDD], and dementia with Lewy bodies [DLB]) from atypical parkinsonian syndromes (APS) with high diagnostic accuracy^8,9^. Alternatively, the involvement of the autonomous nervous system can be assessed by imaging postganglionic cardiac innervation with MIBG and planar scintigraphy. Cardiac MIBG uptake is markedly reduced in patients with LBD when compared to healthy controls and APS^10^. Unlike with FDG PET, a further differentiation between the APS subgroups MSA, progressive supranuclear palsy (PSP), and corticobasal syndrome (CBS) is not possible with MIBG scintigraphy. However, the preferred method is unclear and a direct comparison of both methods is still lacking.

Against this background, the present multicenter study compares the performance of FDG PET and myocardial MIBG scintigraphy in the differential diagnosis of neurodegenerative parkinsonism in the same patient population. Although reduced myocardial innervation is a common finding in LBD in contrast to preserved innervation in non-LBD, most available data on MIBG scintigraphy addresses the differentiation between PD and MSA. Thus, the present study addresses both aforementioned distinctions.

## Results

### Patient characteristics

Clinical consensus rating revealed a final follow-up diagnosis of LBD in 19 patients (PD, *n* = 17; PDD, *n* = 1, DLB, *n* = 1), and a diagnosis of APS in 31 patients (MSA, *n* = 28; PSP, *n* = 3). Three patients had another diagnosis than neurodegenerative parkinsonism (i.e. drug-induced parkinsonism, *n* = 2, normal pressure hydrocephalus, *n* = 1). In one patient the lack of sufficient clinical data precluded a final diagnosis. This subject was excluded from statistical analyses, whereas no patient had to be excluded because of interfering medication or corrupted imaging data. All patients who underwent dopamine transporter SPECT during the time course of clinical observation showed a pathological scan result, confirming neurodegeneration (clinical diagnosis of LBD in *n* = 12 and non-LBD in *n* = 11). There were no group differences in terms of sex, age at first imaging, and time of clinical observation between patients with LBD and non-LBD as well as between patients with PD and MSA. However, not unexpectedly, symptom duration at first imaging was longer in patients with LBD (vs. non-LBD; *p* = 0.001) and PD (vs. MSA; *p* < 0.001), respectively (Table 1).

**Table 1:** Demographic characteristics of patient groups.

Analysis	Patient group (clinical diagnosis)	Sex (female/male)	Age at first imaging (years)	Symptom duration at first imaging (years)	Time of clinical observation (years)
A	LBD	6/13	61.3 ± 9.5	9.3 ± 6.8*	2.3 ± 2.5
	Non-LBD	14/20	64.6 ± 9.3	3.9 ± 3.2*	1.4 ± 2.2
B	PD	6/11	60.7 ± 9.5	9.6 ± 7.2**	2.5 ± 2.6
	MSA	12/16	64.0 ± 9.1	3.7 ± 2.6**	1.6 ± 2.3

Data are presented as mean value ± SD. Diseases without Lewy bodies: MSA, *n* = 28; progressive supranuclear palsy, *n* = 3; drug-induced parkinsonism, *n* = 2; normal pressure hydrocephalus, *n* = 1.

*LBD* Lewy body disease, *non-LBD* disease without Lewy bodies, *PD* Parkinson’s disease, *MSA* multiple system atrophy.

*p = 0.001, **p < 0.001, two-sample *t* test.

### Discriminative analysis of FDG PET and MIBG scintigraphy

**Fig. 1:**
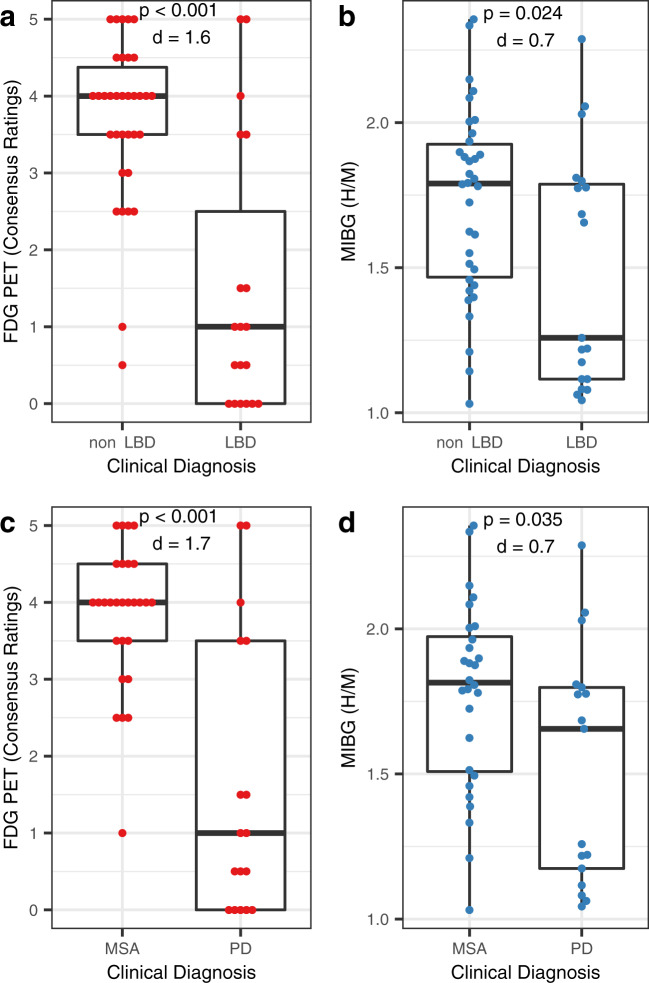
Boxplots for FDG PET and MIBG scintigraphy. **a** FDG PET and **b** MIBG scintigraphy in the differentiation of Lewy body diseases and diseases without Lewy bodies. **c** FDG PET and **d** MIBG scintigraphy in the differentiation of multiple system atrophy and Parkinson’s disease. Center line, median; box limits, upper and lower quartiles; whiskers, 1.5 × interquartile range; *p* values refer to Mann–Whitney *U*-test, d values to Cohen’s effect size. *non LBD* disease without Lewy bodies, *LBD* Lewy body disease, *H/M* heart-to-mediastinum ratio, *MSA* multiple system atrophy, *PD* Parkinson’s disease.

**Fig. 2:**
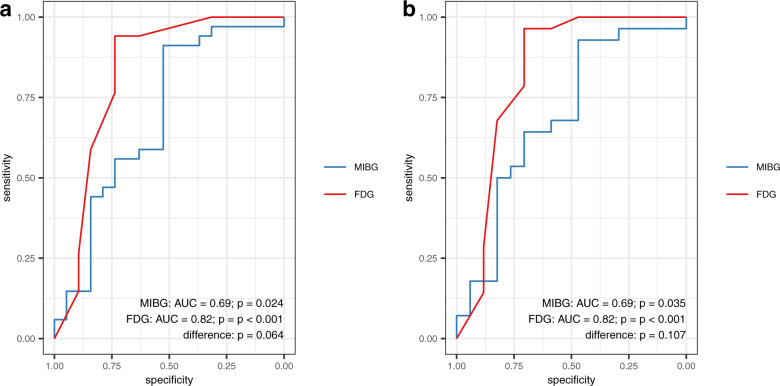
Receiver operating characteristic curves. **a** Differentiation of Lewy body disease from diseases without Lewy bodies and **b** differentiation of Parkinson’s disease and multiple system atrophy. *MIBG* MIBG scintigraphy, *FDG* FDG positron emission tomography, *AUC* Area under the receiver operating characteristic curve.

Inter-rater agreement was very high to excellent (FDG score: weighted *κ* = 0.85 [95%-confidence interval (CI): 0.85–0.85]; FDG second-level diagnosis: *κ* = 0.80 [0.65–0.94]; MIBG H/M: ICC *r* = 0.99 [0.98–0.99]). FDG PET separated non-LBD from LBD and MSA from PD with a ROC AUC of 0.82 (0.68–0.97) and 0.82 (0.67–0.98), respectively, and with large effect sizes (LBD vs. non-LBD: *d* = 1.6; PD vs. MSA: *d* = 1.7; Figs. 1a, c and 2). For MIBG scintigraphy, the ROC AUC for differentiation between LBD and non-LBD and between PD and MSA were notably smaller (0.69 [0.53–0.85] and 0.69 [0.52–0.86], respectively; Fig. 2), yielding medium to large effect sizes (LBD vs. non-LBD: *d* = 0.7; PD vs. MSA *d* = 0.7; Figs. 1b, d). For differentiating LBD vs. non-LBD, the difference between the ROC AUC for FDG PET and MIBG showed a tendency towards statistical significance (*p* = 0.06; Fig. 2a). The difference between the ROC AUC was comparable for the differentiation of PD vs. MSA, but missed trend level significance in the smaller patient sample (*p* = 0.11; Fig. 2b). Interestingly, the ROC AUC for FDG PET was significantly greater than that for MIBG scintigraphy for differentiation of LBD vs. non-LBD for rater #1, who has more experience in FDG PET readings (0.83 [0.69–0.96] vs. 0.69, *p* = 0.04; trend level for PD vs. MSA: 0.83 [0.68–0.98] vs. 0.69 *p* = 0.07). Further sub-analyses after the exclusion of three patients with non-neurodegenerative parkinsonism (i.e. LBD vs. APS) and two additional patients clinically rated as PDD/DLB (i.e. PD vs. APS) confirmed overall findings without substantial changes of the ROC AUC of FDG PET (LBD vs. APS: 0.84 [0.70–0.98]; PD vs. APS: 0.82 [0.67–0.98]) and MIBG scintigraphy (LBD vs. APS: 0.71 [0.55–0.87]; PD vs APS: 0.68 [0.51–0.85]; FDG PET vs. MIBG scintigraphy, each *p* = 0.08; for more details see Supplementary Table 1).

**Fig. 3:**
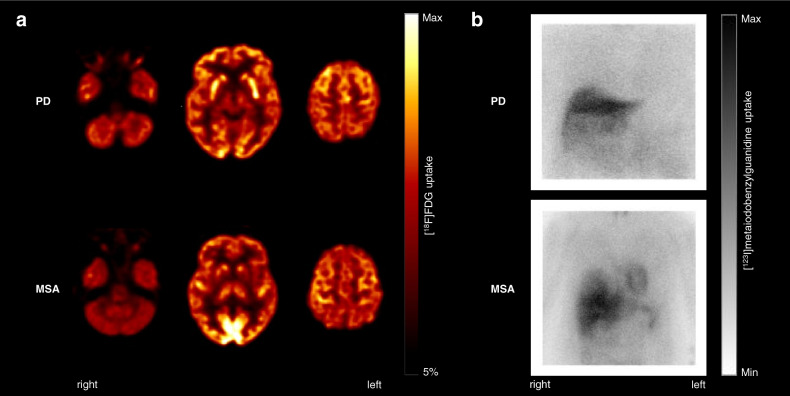
Typical FDG PET and MIBG scintigraphy findings in individual patients with Parkinson’s disease and multiple system atrophy. **a** Spatially normalized transaxial FDG PET slices at the level of cerebellum, basal ganglia, and dorsal frontoparietal cortex. Datasets were thresholded for optimal display. **b** Anterior view of planar MIBG scintigraphy. The patient with PD shows a typical relative hypermetabolism of the bilateral putamen and no cardiac MIBG uptake. The MSA patient is characterized by hypometabolism of the putamen (particularly on the left side), the bilateral cerebellum, and a preserved cardiac uptake on MIBG scintigraphy. *PD* Parkinson’s disease, *MSA* multiple system atrophy.

Optimal thresholds for delineating non-LBD from LBD and MSA from PD were >2.0 for FDG PET (LBD vs. non-LBD: *J* = 0.68; PD vs. MSA: *J* = 0.67) and >1.3 for MIBG scintigraphy (LBD vs. non-LBD: *J* = 0.44; PD vs. MSA: *J* = 0.40), leading to a comparable sensitivity of 94% (85–100%) and 96% (75–100%) for FDG PET and 91% (47–100%) and 93% (46–100%) for MIBG scintigraphy, respectively. In contrast, the specificity was notably higher for FDG PET (74% [53–95%] and 71% [47–94%], respectively) than for MIBG scintigraphy (53% [32–95%] and 47% [29–94%], respectively). Corresponding positive and negative predictive values and positive and negative likelihood ratios for the present patient population are listed in Table 2. These also suggest superiority of FDG PET over MIBG scintigraphy (we refrained from additional pairwise statistical comparisons given the preceding comparisons of ROC analyses). Typical findings for both modalities are shown in Fig. 3.

**Table 2:** Discriminative measures of FDG PET and MIBG scintigraphy.

Analysis	Modality	ROC AUC	Sensitivity	Specificity	PPV	NPV	LR+	LR−
A	MIBG	0.69 (0.53–0.85)	0.91 (0.47–1.00)	0.53 (0.32–0.95)	0.78 (0.71–0.94)	0.77 (0.47–1.00)	1.92 (1.26–3.53)	0.17 (0.00–0.45)
	FDG	0.82 (0.68–0.97)	0.94 (0.82–1.00)	0.74 (0.53–0.95)	0.86 (0.78–0.96)	0.88 (0.70–1.00)	3.58 (1.99–14.06)	0.08 (0.00–0.22)
B	MIBG	0.69 (0.52–0.86)	0.93 (0.46–1.00)	0.47 (0.29–0.94)	0.74 (0.69–0.95)	0.80 (0.48–1.00)	1.75 (1.17–3.10)	0.15 (0.04–0.45)
	FDG	0.82 (0.67–0.98)	0.96 (0.75–1.00)	0.71 (0.47–0.94)	0.84 (0.76–0.96)	0.92 (0.68–1.00)	3.28 (1.72–8.44)	0.05 (0.00-0.18)

Values in parentheses give 95% confidence intervals.

*A* analysis for the differentiation of Lewy body diseases versus diseases without Lewy bodies, *B* analysis for the differentiation of Parkinson’s disease versus multiple system atrophy, *MIBG* MIBG scintigraphy, *FDG* FDG positron emission tomography, *ROC AUC* area under the receiver operating characteristic curve, *PPV* positive predictive value, *NPV* negative predictive value, *LR*+ positive likelihood ratio, *LR−* negative likelihood ratio.

Using the aforementioned thresholds, five and two FDG PET ratings were classified respectively as false-positive (i.e. imaging diagnosis of non-LBD in case of clinical LBD) and false-negative (i.e. imaging diagnosis of LBD in case of clinical non-LBD). In the case of MIBG scintigraphy, there were nine false-positive and three false-negative ratings. Of note, in four false-positive cases and one false-negative case, the FDG PET and MIBG scintigraphy ratings were congruent.

Finally, within the groups of patients with APS according to the clinical follow-up diagnosis (*n* = 31), FDG PET ratings indicated MSA (*n* = 26), PSP (*n* = 3), CBS (*n* = 1), or PD (*n* = 1). The FDG PET ratings agreed with the clinical follow-up diagnosis in 87% of cases (*χ*² = 12.37, *p* < 0.01) with the exceptions being *n* = 3 MSA and *n* = 1 PSP by clinical diagnosis that were diagnosed with PD, PSP and CBS, and MSA by FDG PET ratings, respectively.

## Discussion

The present study suggests that FDG PET provides higher diagnostic accuracy than MIBG scintigraphy for the differentiation between LBD and non-LBD as well as between PD and MSA using the blinded follow-up diagnosis as reference. The differences between both modalities did not reach statistical significance (*p* = 0.06 and *p* = 0.11, respectively), although FDG PET performed significantly better than MIBG scintigraphy when considering the individual ratings of the more experienced rater #1 (LBD vs. non-LBD, *p* = 0.04; PD vs. MSA, *p* = 0.07).

These results extend prior studies by providing a within-subject comparison of FDG PET and MIBG scintigraphy and match recent, independent meta-analyses suggesting modest superiority of FDG PET^7,10^. However, specificity of both modalities in the present study were lower than previously described: for delineating non-LBD from LBD, the calculated sensitivity of FDG PET (94%) was in the upper range of the expected sensitivity (91%, 95%-CI: 72–98%) based on a recent meta-analysis^7^, whereas the specificity (74%) was at the lower limit of the expected range (91%; 95%-CI: 70–98%). MIBG scintigraphy discriminated both groups with 91% sensitivity and 53% specificity, which is comparable sensitive (83%; 95%-CI: 60–94%) but less specific (89%; 95%-CI: 82–95%) compared to the values reported by a recent meta-analysis^10^ (note that figures from this reference were adjusted to meet the present definition of positive [non-LBD] and negative cases [LBD]). Aside from the actual selection of the diagnostic cut-off values, the difference between aforementioned studies and the present study may also be related to the retrospective design of our study. We included only patients investigated with both FDG PET and MIBG scintigraphy for diagnostic purpose in clinical routine. In the participating tertiary reference centers, patients fulfilling this prerequisite are usually highly complex cases with clinically inconclusive findings. Although the disease course revealed a clear follow-up diagnosis in most of the patients, some clinical uncertainty remained in some cases until the last contact. Likewise, it can be expected that causes of clinical uncertainty (e.g., comorbid conditions, very early or advanced disease stage) may also translate into less typical imaging findings. Thus, both clinical and imaging uncertainty may result in less optimal estimates of the diagnostic accuracy in the present study compared to earlier studies. The present results and their statistical interpretation are obviously crucially dependent on the actual sample size (e.g., see identical differences of ROC AUCs for LBD vs. non-LBD [*n* = 53] and PD vs. MSA [*n* = 45], reaching a statistical trend level only for the slightly larger sample, or the only marginally higher AUC of rater #1 [0.83], implying a significant difference, as apposed to the consensus read [0.82]). Thus, confirmation of the present results by a larger patient sample is advisable. Of note, the use of different scales (i.e. continuous for MIBG scintigraphy and ordinal for FDG PET) may have introduced a negative bias concerning the diagnostic performance of FDG PET, since an ordinal scale with few steps may result in an underestimation of the AUC^11^. By calculating the average score for both raters, however, the consensus rating provided an 11-step ordinal scale, which almost approximates a continuous scale so that the effect is probably negligible.

Besides the more favorable diagnostic performance, an additional advantage of FDG PET over MIBG scintigraphy is the possibility to further differentiate between the APS^7^. This also applies to the present study, where 87% of patients with APS were correctly classified as either MSA or PSP by FDG PET. Although not contemplated by the present study, FDG PET may also be used to detect cortical involvement in PD and predict future cognitive decline^12–14^. This may be of tremendous prognostic importance^15^ and of particular clinical relevance, e.g., in the context of deep brain stimulation, where the risk of cognitive deterioration should be estimated before surgery^16,17^.

MIBG scintigraphy has several handicaps in clinical practice. First, its reliability is limited due to the fact that patients with early PD (Hoehn & Yahr 1-2) show a normal MIBG uptake in more than 25% of cases^18^ and up to 30% of patients with MSA may show an impaired cardiac innervation^19^. In combination with neuropathological findings (i.e., alpha-synuclein related pathology in thoracic sympathetic ganglia in patients with advanced MSA^20^), the latter suggests overlapping mechanisms of peripheral denervation in both diseases and thus a conceptual limitation of cardiac MIBG scintigraphy. Second, cardiac denervation is not specific for LBD but may also be present in patients with congestive heart failure, ischemic heart disease, or diabetic neuropathy^21,22^. These conditions hamper proper differential diagnosis and need to be ruled out before MIBG scintigraphy, which might require additional diagnostic procedures and restricts the target population of MIBG scintigraphy, especially in elderly patients. Lastly, several mechanisms of interference of various classes of drugs with MIBG exist and can influence cardiac tracer uptake. Although standard dopaminergic treatment is not critical, several common cardiovascular agents, antipsychotics, and antidepressants have interactions and need to be discontinued according to their half-life up to 4 weeks prior to imaging^23,24^. In our sample, a diligent review of patient charts ruled out that patients had relevant comorbidities or received any interfering medication at time point of imaging. Therefore, we can exclude these confounders. However, if not considered carefully, the diagnostic accuracy of MIBG scintigraphy might be even lower than currently recorded.

Considering FDG PET, earlier studies indicated that its diagnostic accuracy is not relevantly affected by disease-stage and common medications^7^, while comorbidities with morphological brain alterations (e.g., gross atrophy, ischemic lesions) may complicate image interpretation. Furthermore, FDG PET might have one disadvantage towards MIBG scintigraphy in certain clinical settings: If disease-specific metabolic patterns are identified by visual reads and not automated classification methods^7^, the diagnostic accuracy of FDG PET may rely on the experience of the reader. Correct ROI placement on planar MIBG images, in turn, is easier to learn and makes image evaluation less susceptible to the reader’s experience than is the case for FDG PET. This is also suggested by the present study, in which the independent rating of the more experienced investigator indicated statistically significant superiority of FDG PET, which was not the case for the less experienced investigator. However, the actual difference between both readers was small (see above), which we attribute to the auxiliary use of easy-to-interpret voxel-based statistical analyses. With the support of three-dimensional stereotactic surface projections or single-subject SPM analyses, non-expert investigators can achieve a diagnostic accuracy, which is similar to the performance of experts^25,26^.

The retrospective nature of the present study implies an inherent risk of bias. We addressed this by creating clinical vignettes containing comprehensive clinical data in line with current diagnostic criteria. These were filled in by the same expert at every center, which were then evaluated by two blinded movement disorder specialists in consensus. However, having received both imaging techniques, the patient populations probably entail a selection bias towards complex cases, which probably leads to more conservative estimates of the diagnostic accuracies of the enrolled methods (see above). Of note, the order of both examinations was roughly balanced which argues against a possible bias. Another limitation related to the retrospective design is the time gap between scans. However, the exclusion of six subjects with a time gap >3 months did not reveal a relevant effect on the study outcome (data not shown in detail). Another potential source of bias is the use of different collimators at each study center, what we accounted for by using a linear correction method^27,28^. However, separate ROC analyses for each center are in agreement with the overall results suggesting that the use of different collimators did not have a major impact on the results (see Supplementary Table 2).

Given the limited accuracy of the clinical diagnosis of LBD and APS as illustrated by clinicopathological studies^29–33^, the reliance on the clinical diagnosis represents another limitation. We tried to minimize the risk of incorrect clinical diagnosis by including all accessible patient information into the vignettes. However, the observation that a substantial fraction of incorrect FDG PET and MIBG scintigraphy ratings occurred unanimously (in particular, see false-positive cases in Fig. 1 with highly confident FDG PET ratings and high H/M ratios) may suggest that actually the clinical diagnosis was false-negative in these FDG PET and MIBG scintigraphy-positive cases. This underlines the general need for future prospective studies including the post mortem histopathological diagnose as reference in the field of imaging in parkinsonian syndromes^7^.

Taken together, in the present study involving a clinically challenging patient population, FDG PET and MIBG scintigraphy correctly classified approximately four out of five and two out of three patients, respectively. Albeit this notable difference did not attain statistical difference, we consider this finding clinically relevant and suggest that FDG PET, which also allows for subgrouping of APS, should be preferred.

## Methods

### Patients

The present study represents a retrospective analysis of imaging data from two university hospitals. The patients came from three tertiary referral centers specialized in movement disorders. We screened the records of the Department of Nuclear Medicine, Medical Center - University of Freiburg and the Department of Nuclear Medicine, University Hospital Würzburg between 2012 and 2018 for patients who received both FDG PET and MIBG scintigraphy. Patients who underwent both imaging techniques for the differential diagnosis of LBD and APS, and had a clinical follow-up ≥1 year were eligible for this study (inclusion criteria; in total *n* = 54; Freiburg *n* = 27; Würzburg *n* = 27). Incomplete clinical data, interfering medication at the time of MIBG scintigraphy and/or corrupted image data served as exclusion criteria. Forty patients (74.1%) completed both scans within one week and forty-eight patients (88.9%) within three months. The mean time between both scans was 10 weeks ±34 weeks.

The clinical follow-up diagnosis served as reference standard. To standardize this process across all three referral sites, one movement disorder specialist (N.S.) blinded to FDG PET and MIBG scintigraphy reviewed all available information from the first to the last patient contact of each subject (i.e., time of clinical observation). The information included prior patient history and clinical follow-up (≥1 year), all physical and neurological examinations, clinical charts (incl. past and present medications), tilt-table test, Schellong test, urodynamic study, and imaging results other than FDG PET and MIBG scintigraphy (i.e. transcranial sonography, MRI, CT, and dopamine transporter SPECT), and was incorporated into a standardized clinical case vignette derived from the current diagnostic criteria of LBD and APS^3–6,34^. In analogy to an earlier study^35^, we developed the structure of the vignette for this study specifically, which contained sections with demographic information, symptomatology, diagnostic findings, therapy, and disease course, covering all relevant information before and after imaging procedures (see Supplementary Data 1 for the template of the vignette). Of note, results of FDG PET and MIBG scintigraphy, as well as the diagnosis of the treating neurologists were not recorded. Two board-certified neurologists and movement disorder specialists (W.H.J. and I.U.I.) independently evaluated the clinical vignettes of all patients according to current diagnostic criteria^3–6,34^. The rating contained two consecutive levels: first, raters classified each vignette as indicative for LBD, APS, or other. Second, raters sub-classified patients with LBD and APS as PD, PDD, or DLB and PSP, MSA, or CBS, respectively. After independent assessment of all vignettes, both raters reached a consensus follow-up diagnosis (i.e. the reference standard).

### FDG PET

Patients were examined in medication-on state (if applicable) since the effect of dopaminergic medication on FDG PET is considered negligible^7^. Scans were obtained either on a Biograph mCT 64 (Siemens Medical Solutions, Knoxville, TN; *n* = 27, mean ± SD injected dose, 204 ± 10 MBq FDG) or a Philips Gemini TrueFlight 64 integrated PET/CT system (TF64, Philips, Eindhoven, The Netherlands; *n* = 27, 214 ± 8 MBq FDG). PET emission data were acquired 30 min post-injection on the Biograph mCT64 and 50 min post-injection on the Philips Gemini TrueFlight in 3-dimensional mode for 10 min in accordance with current procedural guidelines for FDG brain imaging^36^. PET data were reconstructed iteratively and fully corrected for randoms, scatters, and photon attenuation using a low-dose CT for attenuation correction and vendor-specific reconstructions methods.

We performed FDG PET data analysis with an in-house pipeline written in MATLAB (The MathWorks, Inc.) and employing statistical parametric mapping routines (SPM12, Wellcome Department of Cognitive Neurology, University College, London). We spatially normalized the scans to an FDG PET template image in Montreal Neurological Institute (MNI) brain space, followed by proportional scaling of voxel-wise FDG uptake to mean global brain parenchyma FDG uptake. Two board-certified nuclear medicine physicians (P.T.M. and C.L.), blinded to clinical information, then independently rated FDG PET scans of all 54 patients by using 30 transaxial slices (4.5 mm thickness) covering the entire brain. Slices were displayed in a standardized fashion (maximum adjusted for optimal display, minimum set to 5% of maximum, monochrome “hot metal” color scale). As supportive analyses, readers had access to three-dimensional stereotactic surface projections (3D SSP; Neurostat software) displaying the deviation of each individual’s regional cerebral FDG uptake from age-matched healthy controls (color-coded *Z* score 0–7; decreases only)^37^ and the results of single-subject SPM analyses showing significantly (*p* < 0.05, *k* > 50 voxels, no correction for multiple comparisons) hyper- and hypometabolic regions in individual scan compared to healthy controls of comparable ages examined with identical scanners (Gemini TrueFlight: 21 females/14 males, age: 77.6 ± 5.9 years; Biograph mCT: 5 females/5 males, age: 61.9 ± 14.4 years)^25^. The raters interpreted FDG PET scans in two consecutive levels^9^ based on previously published disease-specific patterns of regional cerebral glucose metabolism^7^:

1. First level: readers classified each scan as indicative of LBD or APS using a 6-step score (0/1/2: definite/probable/possible LBD; 3/4/5: possible/probable/definite APS).
2. Second level: readers categorized APS-positive scans (scores 3–5 at first level) as being indicative of MSA, PSP, or CBS.

After independent evaluation of all scans, the raters averaged the scores (yielding an 11-step scale) and reached a consensus second-level diagnosis.

### MIBG scintigraphy

We reviewed patient charts to rule out medication possibly interfering with MIBG scintigraphy^23,38^. MIBG studies were acquired on a dual-headed gamma camera (E.CAM [Siemens Healthineers, Erlangen, Germany], *n* = 10) and two dual-headed SPECT/CT systems (Symbia T2 [Siemens Healthineers, Erlangen, Germany], *n* = 27; Brightview XCT [Philips Medical Systems Inc. Cleveland, OH], *n* = 17). Systems were equipped with either a low-energy high resolution collimator (LEHR; E.CAM and Brightview XCT; *n* = 27, 192 ± 27 MBq MIBG) or a medium-energy low penetration collimator (MELP; Symbia T2; *n* = 27, 183 ± 7 MBq MIBG). Anterior and posterior planar images were obtained for 10 min at 240 min after injection. Two board-certified nuclear medicine physicians (C.L. and J.B., blinded to clinical information) evaluated MIBG uptake semi-quantitatively by calculating the delayed heart-to-mediastinum ratio (H/M) using the planar anterior images. They independently defined region-of-interests (ROI) of the heart and the mediastinum by manually adjusting a circular ROI to the left ventricle and a rectangular ROI to the upper mediastinum using PMOD Version 3.7 (PMOD Technologies Ltd., Zurich, Switzerland). The H/M of each patient was calculated by dividing the mean counts per pixel in the cardiac ROI by the mean counts per pixel in the mediastinal ROI. We then linearly converted the H/M ratio of the patients who had been investigated on the system with the MELP collimator (*n* = 27) into H/M ratios for LEHR collimators^27,28^ and calculated the mean H/M ratio of both raters for each patient.

### Statistical analysis

We used the statistics software R 3.3.3 [http://www.R-project.org/] for statistical analyses. Inter-rater agreement was evaluated with the intra-class-correlation coefficient (ICC) and Cohen’s (weighted) kappa (*κ*) as applicable (R package ‘psych’, version 1.8.12). Between-group differences were assessed using two-sample *t* test, Mann-Whitney *U*-test, and Cohen’s effect size *d* (R package ‘effsize’, version 0.7.4). Receiver operating characteristic (ROC) analyses (R package ‘pROC’, version 1.10.0) were employed to assess and compare the diagnostic performance of the two methods by the area under the ROC curve (AUC). We defined diseases other than LBD (non-LBD; i.e., APS in the vast majority of cases) and MSA, respectively as positive cases for calculating sensitivity and specificity. We selected the thresholds for interpreting FDG PET and MIBG scintigraphy in order to maximize Youden’s *J* (*J* = sensitivity+specificity−1). The association between the clinical follow-up diagnosis and FDG PET ratings was assessed with Pearson’s *χ*² test in the subgroup of APS patients.

### Ethical approval and patient consent

Cardiac MIBG scintigraphy and FDG PET scans were performed as part of the clinical work-up. All patients gave written informed consent to the diagnostic procedures. All procedures performed in humans were in accordance with the principles of the Declaration of Helsinki and its later amendments or comparable ethical standards. The retrospective analysis was approved by the local institutional review boards of the University Hospital Freiburg and the Julius-Maximilian-University Würzburg.

### Reporting summary

Further information on research design is available in the Nature Research Reporting Summary linked to this article.

## Supplementary information

Supplementary Files

Reporting Summary Checklist

## Data Availability

The data that support the findings of this study are available from the corresponding author upon reasonable request.

## Code availability

Data were analyzed using statistics software R 3.3.3 [http://www.R-project.org/] and the R packages ‘psych’ (version 1.8.12), ‘effsize’ (version 0.7.4), and ‘pROC’ (version 1.10.0).
