# Supplementary material for: Differential diagnosis of parkinsonism: a head-to-head comparison of FDG PET and MIBG scintigraphy
Source: NPJ Parkinsons Dis. 2020 Dec 11;6:39. doi: 10.1038/s41531-020-00141-y (PMC7733458; doi:10.1038/s41531-020-00141-y)
Supplement: Supplementary file 1 — Supplementary Files [file 41531_2020_141_MOESM1_ESM.pdf]

SUPPLEMENTARY FILES

Supplementary Table 1 Discriminative measures of FDG PET and MIBG scintigraphy

Analysis	Modality	ROC AUC	Sensitivity	Specificity	PPV	NPV	LR+	LR-
C	MIBG	0.71 (0.55-0.87)	0.94 (0.48-1.00)	0.53 (0.327-0.95)	0.76 (0.70-0.95)	0.83 (0.51-1.00)	1.97 (1.28-3.54)	0.12 (0.00-0.38)
	FDG	0.84 (0.70-0.98)	0.97 (0.87-1.00)	0.74 (0.53-0.95)	0.86 (0.77-0.96)	0.93 (0.76-1.00)	3.68 (1.88-9.27)	0.04 (0.00-0.16)
D	MIBG	0.68 (0.51-0.85)	0.94 (0.45-1.00)	0.47 (0.29-0.94)	0.76 (0.71-0.95)	0.80 (0.45-1.00)	1.77 (1.18-3.09)	0.15 (0.04-0.45)
	FDG	0.82 (0.67-0.98)	0.97 (0.77-1.00)	0.71 (0.47-0.94)	0.86 (0.78-0.97)	0.92 (0.67-1.00)	3.29 (1.88-12.66)	0.05 (0.00-0.18)

Values in parentheses give 95% confidence intervals. C, Analysis for the differentiation of Lewy body diseases versus atypical parkinsonian syndromes; D, analysis for the differentiation of Parkinson's disease versus atypical parkinsonian syndromes. *MIBG* MIBG scintigraphy, *FDG* FDG positron emission tomography, *ROC AUC* area under the receiver operating characteristic curve, *PPV* positive predictive value, *NPV* negative predictive value, *LR+* positive likelihood ratio, *LR-* negative likelihood ratio.

Supplementary Table 2 Discriminative measures of MIBG scintigraphy for each center

Analysis	Center	ROC AUC	Sensitivity	Specificity	PPV	NPV	LR+	LR-
A	both	0.69 (0.53-0.85)	0.91 (0.47-1.00)	0.53 (0.32-0.95)	0.78 (0.71-0.94)	0.77 (0.47-1.00)	1.92 (1.26-3.53)	0.17 (0.00-0.45)
	Freiburg	0.72 (0.49-0.96)	0.90 (0.32-1.00)	0.57 (0.43-1.00)	0.85 (0.81-1.00)	0.67 (0.35-1.00)	2.09 (0.88-4.98)	0.18 (0.04-0.79)
	Würzburg	0.69 (0.48-0.91)	0.93 (0.47-1.00)	0.50 (0.25-1.00)	0.70 (0.63-1.00)	0.86 (0.56-1.00)	1.87 (1.04-3.34)	0.13 (0.02-0.96)

Values in parentheses give 95% confidence intervals. A, Analysis for the differentiation of Lewy body diseases versus diseases without Lewy bodies. *ROC AUC* area under the receiver operating characteristic curve, *PPV* positive predictive value, *NPV* negative predictive value, *LR+* positive likelihood ratio, *LR-* negative likelihood ratio.

Supplementary Data 1 Template of the case vignette for rating of clinical follow-up diagnosis

Gender	*(female / male)*
Year of birth	*(year)*
Year of the last diagnosis:	*(year)*
Age at diagnosis:	*(years)*
Year of first symptoms:	*(year)*
Age at first symptoms:	*(years)*
First contact:	*(year)*
Last contact:	*(year)*
Year of death (if applicable):	*(year)*
General and neurological / psychiatric comorbidities:	
Medication at the time of first clinical contact: *(type, dose)*	
Device-aided therapies: *(type, year, effectiveness)*	
Date FDG PET:	*(mm/yyyy)*
Time from symptom onset until FDG-PET:	*(years)*
Time after FDG-PET in clinical observation:	*(years)*
Date MIBG scintigraphy:	*(mm/yyyy)*
Time from symptom onset until MIBG scintigraphy:	*(years)*
Time after MIBG scintigraphy in clinical observation:	*(years)*
Medication at the time of imaging:<br>*(separated for FDG PET and MIBG scintigraphy, if applicable)*	

Supplementary Data 1 (continuation)

Yes	Feature
☐	(1) L-dopa response at dose *(dose)*
☐	(2) L-dopa response in % *(percentage in UPDRS III score)*
☐	(3) Family history of Parkinson's syndrome
☐	(4) Bradykinesia
☐	(5) Rigidity
☐	(6) Isometric tremor
☐	(7) Rest tremor
☐	(8) Action tremor
☐	(9) Intention tremor
☐	(10) Unilateral disease onset
☐	(11) No highly acute onset
☐	(12) Progression of the disease
☐	(13) Postural instability
☐	(14) Persistent asymmetry
☐	(15) L-Dopa induced hyperkinesia
☐	(16) Hyposmia
☐	(17) Visual hallucinations
☐	(18) Obstipation
☐	(19) Urge incontinence
☐	(20) Depression
☐	(21) Restlessness
☐	(22) REM sleep behaviour disorder (RBD)

Supplementary Data 1 (continuation)

Yes Feature

☐ (23) Ataxia
☐ (24) Gradual deterioration (acute occurrence of new symptoms)
☐ (25) Repeated head trauma
☐ (26) History of encephalitis
☐ (27) Permanent remission of symptoms
☐ (28) Supranuclear gaze palsy
☐ (29) Occurrence of dementia after onset of disease: *(years)*
☐ (30) Impairment of the upper motor neuron
☐ (31) Impairment of the lower motor neuron
☐ (32) Positive pyramidal tract sign (Babinski)
☐ (33) Structural abnormality of the cerebellum (e.g. neoplasm)
☐ (34) Hydrocephalus
☐ (35) Pure unilateral impairment > 3 years
☐ (36) Falls > 1/year in the first 3 years
☐ (37) Parkinsonian features restricted to the lower limbs > 3 years
☐ (38) Cortical sensory loss
☐ (39) Apraxia
☐ (40) Aphasia
☐ (41) Improvement of symptoms after lumbar puncture (if available)
☐ (42) Rapid progression of gait impairment requiring regular use of wheelchair within 5 years of onset
☐ (43) Palilalia
☐ (44) Isolated freezing
☐ (45) Absence of progression of motor symptoms or signs over 5 years
☐ (46) Severe dysphonia within first 5 years
☐ (47) Stiff gait with knee-extended legs
☐ (48) Dyskinesia during L-dopa treatment
☐ (49) Fluctuating cognition
☐ (50) Psychotic symptoms without L-dopa
☐ (51) Psychotic symptoms during L-dopa treatment
☐ (52) Psychotic symptoms during neuroleptic treatment
☐ (53) Autonomic dysfunction
☐ (54) Severe dysphagia within first 5 years
☐ (55) Severe orthostatic dysregulation
☐ (56) Urinary retention
☐ (57) Anterocollis
☐ (58) Contractures
☐ (59) Myoclonia
☐ (60) Alien limb phenomenon
☐ (61) Pathological laughing or crying
☐ (62) Inspiratory stridor
☐ (63) Improvement of symptoms under alcohol consumption
☐ (64) Severe disability at disease onset
☐ (65) Decrease of tremor amplitude during distraction
☐ (66) Polymicromyoclonia

Supplementary Data 1 (continuation)

Diagnostics
Genetic findings, if available: *(year, report)*
Tilt table test, if available: *(year, report)*
Schellong Test, if available: *(year, report)*
Tremoranalysis, if available: *(year, report)*
Electrophysiological examination, if available: *(year, report)*
Transcranial sonography, if available: *(year, report)*
cMRI, if available: *(year, report)*
cCT, if available: *(year, report)*
FP-CIT SPECT, if available: *(year, report)*
Sonographic assessment of urinary retention, if available: *(year, report)*
