# Supplementary material for: Differential diagnosis of parkinsonism: a head-to-head comparison of FDG PET and MIBG scintigraphy
Source: NPJ Parkinsons Dis. 2020 Dec 11;6:39. doi: 10.1038/s41531-020-00141-y (PMC7733458; doi:10.1038/s41531-020-00141-y)
Supplement: Supplementary file 2 — Reporting Summary Checklist [file 41531_2020_141_MOESM2_ESM.pdf]

nature research

Corresponding author(s): DBPR NPJPARKD-00480

Last updated by author(s): Oct 28, 2020

Reporting Summary

Nature Research wishes to improve the reproducibility of the work that we publish. This form provides structure for consistency and transparency in reporting. For further information on Nature Research policies, see our Editorial Policies and the Editorial Policy Checklist.

Statistics

For all statistical analyses, confirm that the following items are present in the figure legend, table legend, main text, or Methods section.

n/a	Confirmed	
☐	☒	The exact sample size (*n*) for each experimental group/condition, given as a discrete number and unit of measurement
☐	☒	A statement on whether measurements were taken from distinct samples or whether the same sample was measured repeatedly
☐	☒	The statistical test(s) used AND whether they are one- or two-sided<br>*Only common tests should be described solely by name; describe more complex techniques in the Methods section.*
☐	☒	A description of all covariates tested
☐	☒	A description of any assumptions or corrections, such as tests of normality and adjustment for multiple comparisons
☐	☒	A full description of the statistical parameters including central tendency (e.g. means) or other basic estimates (e.g. regression coefficient) AND variation (e.g. standard deviation) or associated estimates of uncertainty (e.g. confidence intervals)
☐	☒	For null hypothesis testing, the test statistic (e.g. *F*, *t*, *r*) with confidence intervals, effect sizes, degrees of freedom and *P* value noted<br>*Give P values as exact values whenever suitable.*
☒	☐	For Bayesian analysis, information on the choice of priors and Markov chain Monte Carlo settings
☒	☐	For hierarchical and complex designs, identification of the appropriate level for tests and full reporting of outcomes
☐	☒	Estimates of effect sizes (e.g. Cohen's *d*, Pearson's *r*), indicating how they were calculated

Our web collection on statistics for biologists contains articles on many of the points above.

Software and code

Policy information about availability of computer code

Data collection	No software was used for data collection.
Data analysis	Data were analyzed using statistics software R 3.3.3 [http://www.R-project.org/] and the R packages 'psych' (version 1.8.12), 'effsize' (version 0.7.4), and 'pROC' (version 1.10.0).

For manuscripts utilizing custom algorithms or software that are central to the research but not yet described in published literature, software must be made available to editors and reviewers. We strongly encourage code deposition in a community repository (e.g. GitHub). See the Nature Research guidelines for submitting code & software for further information.

Data

Policy information about availability of data

All manuscripts must include a data availability statement. This statement should provide the following information, where applicable:

- Accession codes, unique identifiers, or web links for publicly available datasets
- A list of figures that have associated raw data
- A description of any restrictions on data availability

The data that support the findings of this study are available from the corresponding author upon reasonable request.

Field-specific reporting

Please select the one below that is the best fit for your research. If you are not sure, read the appropriate sections before making your selection.

☒ Life sciences ☐ Behavioural & social sciences ☐ Ecological, evolutionary & environmental sciences

For a reference copy of the document with all sections, see nature.com/documents/nr-reporting-summary-flat.pdf

Life sciences study design

All studies must disclose on these points even when the disclosure is negative.

Sample size	No sample size-calculation was performed. The study represents a retrospective analysis of imaging data. Patients who underwent FDG PET and MIBG scintigraphy for the differential diagnosis of parkinsonian syndromes, and had a clinical follow-up of more than 1 year were eligible for this study (n = 54).
Data exclusions	Incomplete clinical data (n = 1), the taking of interfering medication at the time of MIBG scintigraphy (n = 0), and corrupted image data were defined as exclusion criteria (n = 0).
Replication	Reproducibility was assured by using consensus ratings (clinical diagnosis; FDG PET reads) or consensus values (MIBG) from multiple raters.
Randomization	No randomization was performed. Patients were not allocated to experimental groups. Instead, group allocation was based on clinical diagnosis.
Blinding	Double-blind design: FDG PET reads and MIBG measurements were blinded against the clinical diagnosis and vice versa.

Reporting for specific materials, systems and methods

We require information from authors about some types of materials, experimental systems and methods used in many studies. Here, indicate whether each material, system or method listed is relevant to your study. If you are not sure if a list item applies to your research, read the appropriate section before selecting a response.

Materials & experimental systems

n/a	Involved in the study	
☒	☐	Antibodies
☒	☐	Eukaryotic cell lines
☒	☐	Palaeontology and archaeology
☒	☐	Animals and other organisms
☐	☒	Human research participants
☒	☐	Clinical data
☒	☐	Dual use research of concern

Methods

n/a	Involved in the study	
☒	☐	ChIP-seq
☒	☐	Flow cytometry
☒	☐	MRI-based neuroimaging

Human research participants

Policy information about studies involving human research participants

Population characteristics	The study aimed to compare the diagnostic performance of two imaging modalities. The methods are used to separate between patients with Lewy body disease (LBD) and patients without LBD (non-LBD). The patients of the two groups showed differences (age, symptom duration, and time of clinical observation), which are related to the clinical diagnosis. The primary outcome of the study is not affected by the group differences.
Recruitment	We screened the records of participating study centers for patients who received both FDG PET and MIBG scintigraphy between 2012 and 2018 at two university hospitals. The retrospective nature of the study implies an inherent risk of bias. Having received both imaging techniques, the patient populations probably entail a selection bias towards complex cases, which may lead to more conservative estimates of the diagnostic accuracies of the enrolled methods. Since the order of the two methods was roughly balanced, there was no considerable bias in favour of one modality.
Ethics oversight	The study was approved by the local institutional review boards of the University Hospital Freiburg and the Julius-Maximilian-University Würzburg.

Note that full information on the approval of the study protocol must also be provided in the manuscript.
